# Pathogenic Variants and Clinical Outcomes in Korean Dilated Cardiomyopathy Patients: Insights From Next-Generation Sequencing

**DOI:** 10.1016/j.jacasi.2025.11.001

**Published:** 2026-03-16

**Authors:** Yu Young Kim, Min Sun Kim, Sun-Hak Lee, Jung Hyun Choi

**Affiliations:** a Department of Internal Medicine, Pusan National University Hospital, Busan, South Korea; b Division of Cardiology, Department of Internal Medicine, Pusan National University Hospital, Pusan National University School of Medicine, Busan, Republic of Korea; c Department of Internal Medicine, Pusan National University School of Medicine, Busan, Republic of Korea

**Keywords:** dilated cardiomyopathy, genetics, heart failure, next-generation sequencing, precision medicine

Dilated cardiomyopathy (DCM) features left ventricular dilation and systolic dysfunction leading to heart failure (HF), arrhythmias, or sudden cardiac death.^1^ Although many cases have a genetic basis, Asian, particularly Korean, data remain limited.^2^ This study examined whether genotype positivity predicts adverse outcomes in Korean DCM cohort.

## Methods

This single-center retrospective study included 60 patients who underwent next-generation sequencing (NGS) for suspected or confirmed DCM at Pusan National University Hospital between November 2017 and September 2024, including both inpatients and outpatients.

DCM was defined by transthoracic echocardiographic findings of left ventricular dilation (left ventricular end-diastolic diameter [LVEDD] >58 mm [men], >52 mm [women], or left ventricular end-diastolic volume index ≥75 mL/m^2^ [men], ≥62 mL/m^2^ [women]) with impaired systolic function (left ventricular ejection fraction [LVEF] <50%) without abnormal loading conditions or coronary artery disease.^3^ Genetic testing was performed per 2022 American Heart Association/American College of Cardiology/Heart Failure Society of America guidelines,^4^ including familial or unexplained early-onset DCM with arrhythmias or conduction abnormalities. Patients who refused genetic testing or received NGS after cardiac death were excluded. Institutional review board approval (No. 2505-002-150).

The NGS date was the index for baseline and follow-up, aligning imaging, biomarker, and genotype data at clinically relevant points including new arrhythmia or unexplained deterioration, when genetic information could guide clinical decisions. Demographics, echocardiographic, and laboratory data within 6 months of the NGS date were analyzed.

Transthoracic echocardiographic was performed per American Society of Echocardiography guidelines,^5^^,^^6^ with LVEF calculated by Simpson’s method and left ventricular global longitudinal strain by vendor-independent software (TomTec, Image Arena 4.6).

Genomic DNA was analyzed using a 174-gene custom cardiomyopathy panel (Celemics Inc) on the Illumina MiSeq platform (Illumina Inc). Variant interpretation followed the 2015 American College of Medical Genetics and Genomics/Association for Molecular Pathology guideline with the standard 5-tier classification (pathogenic, likely pathogenic, variant of uncertain significance, likely benign, benign); pathogenic and likely pathogenic variants were classified as genotype-positive, all others as genotype-negative.

The primary endpoint was a composite of all-cause mortality, HF-related events (hospitalizations, left ventricular assist device [LVAD] implantation, heart transplantation), ventricular tachycardia, or appropriate implantable cardioverter-defibrillator shocks.

Continuous variables were expressed as median (Q1-Q3) and compared with the Mann-Whitney *U* test; categorical variables as counts (%) and compared with the chi-square or Fisher exact test. Event-free survival was analyzed with Kaplan-Meier and log-rank tests. Cox proportional hazards models included genotype status, LVEF, and LVEDD. Proportional hazards assumptions were tested with Schoenfeld residuals; LVEF was modeled as time-dependent. Analyses used R version 4.3.1; *P <* 0.05 was significant.

## Results

A total of 60 patients were included: 15 genotype-positive patients (median age 55.0 years [Q1-Q3: 40.0-59.5 years], 13.3% women) and 45 genotype-negative patients 49.0 years [Q1-Q3: 40.0-57.0 years], 28.9% women). Most were NYHA functional class I-III (98.3%), without significant demographic differences.

Echocardiographic findings were comparable: median LVEF 29.0% (Q1-Q3: 20.0%-33.0%) vs 31.0% (Q1-Q3: 22.0%-34.0%); NT-proBNP 2,807 pg/mL (Q1-Q3: 2,416–4,733 pg/mL) vs 2,334 pg/mL (Q1-Q3: 467-4,087 pg/mL); high-sensitivity troponin I and other laboratory parameters (all *P >* 0.05).

*TTN* variants were most frequent (40.0%), followed by *LMNA* (20.0%), *RBM20* (13.3%), and *MYBPC3*, *TNNT2*, *TBX20*, and *KCNQ1* (each 6.7%).

During median 22.4-month follow-up (Q1-Q3: 13.8-28.9 months], 26 patients (43.3%) experienced adverse events. Composite event rates were significantly higher in genotype-positive patients (66.7% vs 35.6%; *P =* 0.035), with increased but nonsignificant rates of cardiac death (33.3% vs 13.3%) and HF-related events (46.7% vs 22.2%). Among *TTN* carriers (n = 6), 5 experienced events (3 hospitalizations, 2 device implantations [implantable cardioverter-defibrillator, LVAD]); among *LMNA* carriers (n = 3), 2 required LVAD implantation.

**Figure 1:**
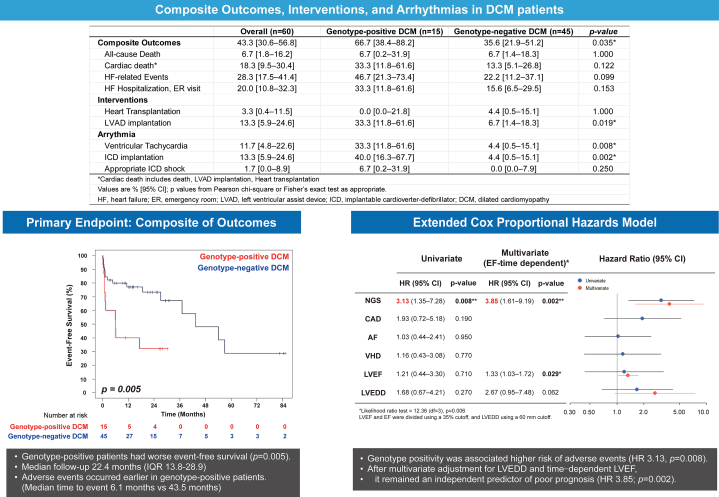
Summary Genotype-Positive Patients Showed Higher Composite Adverse Event and Device-Related Rates. Kaplan-Meier analysis demonstrated shorter event-free survival in genotype-positive patients *(P =* 0.005; median 6.1 months vs 43.5 months). In multivariate Cox analysis, genotype positivity remained an independent predictor of adverse outcomes after adjustment for left ventricular ejection fraction (LVEF) and left ventricular end-diastolic diameter (LVEDD) (HR: 3.85; 95% CI: 1.61-9.19; *P =* 0.002). AF = atrial fibrillation; CAD = coronary artery disease; DCM = dilated cardiomyopathy; HF = heart failure; ICD = implantable cardioverter-defibrillator; LVAD = left ventricular assist device; NGS = next-generation sequencing; VHD = valvular heart disease.

Kaplan-Meier analysis showed shorter event-free survival in genotype-positive patients, median time to event of 6.1 months vs 43.5 months in genotype-negative *(P =* 0.005). On univariate Cox analysis, genotype positivity predicted adverse outcomes (HR: 3.13; 95% CI: 1.35-7.28; *P =* 0.008) and remained significant in multivariable analysis with LVEF and LVEDD (HR 3.54; 95% CI 1.51–8.31; *P =* 0.004), whereas LVEF *(P =* 0.19) and LVEDD *(P =* 0.11) were not. Violations of proportional hazards for LVEF *(P =* 0.011) led to time-dependent modeling, again confirming genotype significance (HR: 3.85; 95% CI: 1.61-9.19; *P =* 0.002) with LVEDD not reaching statistical significance *(P =* 0.062) and an increasing HR for LVEF over time *(P =* 0.029) (Figure 1).

## Discussion

Genotype positivity independently predicted adverse outcomes in Korean DCM patients beyond conventional echocardiographic parameters. LVEF effects were time-dependent, whereas genetic burden conferred persistent risk, underscoring limitations of baseline functional assessment for long-term stratification.^7^^,^^8^

Consistent with Western cohorts,^7^, ^8^, ^9^ *LMNA* and truncating *TTN* variants portended adverse prognosis. Two-thirds of *LMNA* carriers required early LVAD implantation, reflecting the arrhythmogenicity and malignant progression of laminopathies. *TTN* variants, although more heterogeneous, were associated with progressive systolic dysfunction and recurrent HF episodes.

Genotype-positive patients demonstrated elevated composite events without concordant mortality divergence. This likely reflects the short median follow-up of 22.4 months and contemporary therapies delaying mortality. Extended follow-up studies are necessary to definitively evaluate the impact of genotype status on long-term mortality. The increased burden of arrhythmic and hemodynamic complications highlights the need for vigilant monitoring and proactive management.

Variants of uncertain significance were classified as genotype-negative per contemporary standards, a conservative approach that maintains clinical specificity and enables cross-study comparability, yet potentially underestimates genetic burden and may attenuate observed associations. As additional segregation, functional, and population evidence accrues, reclassification may occur, and larger collaborative studies are required to define prognostic implications.

These findings, from a single tertiary center with modest sample size and limited events, demonstrate the independent prognostic value of genetic testing. Although optimal medical therapy was well-implemented across the cohort, the limited sample size and event number precluded inclusion of pharmacotherapy and comorbidity variables in multivariable models to maintain statistical validity and avoid overfitting. Nevertheless, genotype status maintained independent prognostic discrimination. Cardiac magnetic resonance data were limited and therefore excluded given nonstandardized acquisition and substantial missing observations. Larger prospective multicenter studies with adequate statistical power are warranted to enable comprehensive evaluation of medication effects and comorbidity interactions alongside standardized imaging and extended surveillance.

In Korea, implementation of genetic testing remains limited by inadequate insurance coverage, inconsistent interpretation, and scarce counseling resources. National strategies require multidisciplinary services, broader reimbursement, and population-specific databases.

## Funding Support and Author Disclosures

The authors have reported that they have no relationships relevant to the contents of this paper to disclose.

## References

[1] Fatkin D., Huttner I.G., Kovacic J.C. (2019). Precision medicine in the management of dilated cardiomyopathy: JACC state-of-the-art review. J Am Coll Cardiol.

[2] Kim K.H., Pereira N.L. (2021). Genetics of cardiomyopathy: clinical and mechanistic implications for heart failure. Korean Circ J.

[3] McDonagh T.A., Aguilera B., Albert A. (2023). 2023 ESC guidelines for the management of cardiomyopathies: developed by the Task Force on the Management of Cardiomyopathies of the European Society of Cardiology (ESC). Eur Heart J.

[4] Heidenreich P.A., Bozkurt B., Aguilar D. (2022). 2022 AHA/ACC/HFSA guideline for the management of heart failure: a report of the American College of Cardiology/American Heart Association Joint Committee on Clinical Practice Guidelines. J Am Coll Cardiol.

[5] Lang R.M., Badano L.P., Mor-Avi V. (2015). Recommendations for cardiac chamber quantification by echocardiography in adults: an update from the American Society of Echocardiography and the European Association of Cardiovascular Imaging. Eur Heart J Cardiovasc Imaging.

[6] Mor-Avi V., Lang R.M., Badano L.P. (2011). Current and evolving echocardiographic techniques for the quantitative evaluation of cardiac mechanics: ASE/EAE consensus statement on methodology and indications endorsed by the Japanese Society of Echocardiography. J Am Soc Echocardiogr.

[7] Herman D.S., Lam L., Taylor M.R. (2012). Truncations of titin causing dilated cardiomyopathy. N Engl J Med.

[8] Jordan E., Peterson L., Ai T. (2021). Evidence-based assessment of genes in dilated cardiomyopathy. Circulation.

[9] Ferradini V., Cosma J., Romeo F. (2021). Clinical features of LMNA-related cardiomyopathy in 18 patients and characterization of 2 novel variants. J Clin Med.

